# Rhino-orbito-cerebral invasive fungal sinusitis associated with COVID-19 infection in a malnurished child

**DOI:** 10.1186/s43054-022-00152-9

**Published:** 2023-01-04

**Authors:** Mallesh Kariyappa, Ashray Sudarshan Patel, K Dhanalakshmi, B Dakshayani

**Affiliations:** Department of Pediatrics, Vani Villas Hospital, Bangalore Medical College and Research Hospital, Kalasipalyam, Bangalore, India

**Keywords:** COVID-19, Rhino-cerebral, Invasive fungal infection, Orbital cellulitis

## Abstract

**Background:**

Corona virus disease has been associated with a wide variety of fungal and bacterial co-infections. These secondary infections could be due to the irrational use of antibiotics, immunosuppressive therapy, pre-existing co morbidities, and immune modulator effects of the virus. But here, we report a very rare occurring of rhino-orbito-cerebral invasive fungal sinusitis in a malnourished child and no other co morbidities.

**Case presentation:**

This is a case of a 6-year-old boy with severe thinness and no other co-morbidities, with mild COVID-19 infection, during the course of illness developed rhino-orbito-cerebral invasive fungal sinusitis. The child’s mother had COVID-19 1 week prior to child’s illness. The child then developed fever followed by headache. The child reported to hospital on seventh day of illness and RTPCR for COVID-19, turned positive. The child’s vitals were stable and maintaining saturation. Child was being treated with supplements and symptomatic treatment for fever. On his second day of stay at hospital, he started to develop gradually progressive left-sided peri-orbital swelling. Due to the association of COVID-19 with fungal infection, child was started on AMPHOTERICIN-B and given for 4 days and referred to a higher center for further management. Radiological imaging was suggestive of rhino-sinusitis with orbital cellulitis with meningeal enhancement suggestive of fungal etiology. Debridement was done, child was adequately treated with anti-fungal, and the child showed significant improvement along with radiological clearing.

**Conclusion:**

Invasive fungal infection can occur in association with COVID-19 among malnourished pediatric age groups with no other comorbidities.

## Background

The coronavirus disease 2019 infection is caused by the novel severe acute respiratory syndrome corona virus 2 [1]. In the pediatric age group, the disease may be associated with a wide range of disease patterns, ranging from asymptomatic infection to critical disease progressing to ARDS [2]. The occurrence of a multi-system inflammatory syndrome in children is also known, which was initially named as “Kawasaki like-disease” and then renamed pediatric multi-system inflammatory syndrome (PIMS) [3]. A wide range of bacterial and fungal co-infections may exist in COVID-19 patients either as a part of hospital acquired infections or those with pre-existing comorbidities [4, 5].

Among children, the most common cause of invasive fungal infection includes Aspergillus species, followed by Candida and Mucormycosis [6]. Acute invasive fungal rhino-sinusitis is an opportunistic infection that occurs mostly in immunocompromised patients [7]. There are increasing reports of invasive fungal infection associated with coronavirus disease especially in India, amongst people with various co-morbidities [8].

Very few cases have been reported in the pediatric age group with no associated co morbidities. Here, we report a case of child rhino-orbito-cerebral invasive fungal sinusitis associated with coronavirus disease, in a malnourished child. Similar such cases were reported by Turbin et al. among two adolescents with COVID-19 [9].

## Case presentation (Fig. 1)

**Fig. 1:**
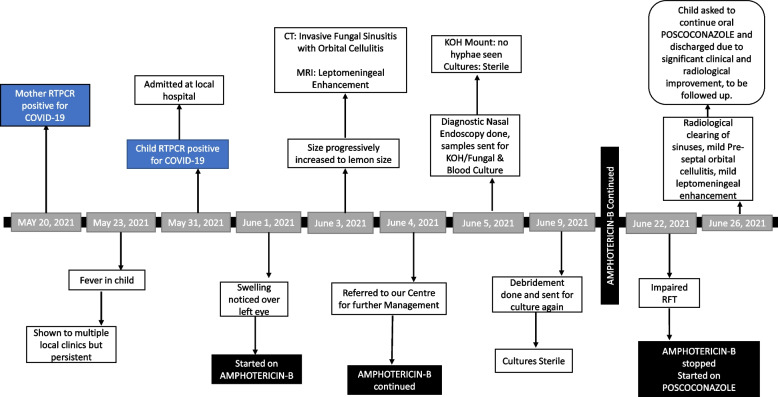
Timeline of the events

A 6-year-old child hailing from a region of high incidence of COVID 19, from a farming background, came to the hospital with complaints of fever from 1 week. One week prior to the illness mother was tested positive for COVID-19. The child had significant exposure to mother. RTPCR for the child was positive. The child was admitted along with the mother in the hospital. At admission, the child was active and had no difficulty in breathing. On examination, vitals were stable, maintaining saturation, and systemic examination was within normal limits. There was no history of any past hospitalisation for any significant illness in the child and had been healthy since birth. So the child was taken to be Mild COVID-19 illness and was being given symptomatic treatment along with supplements. No steroids were being given .

**Fig. 2:**
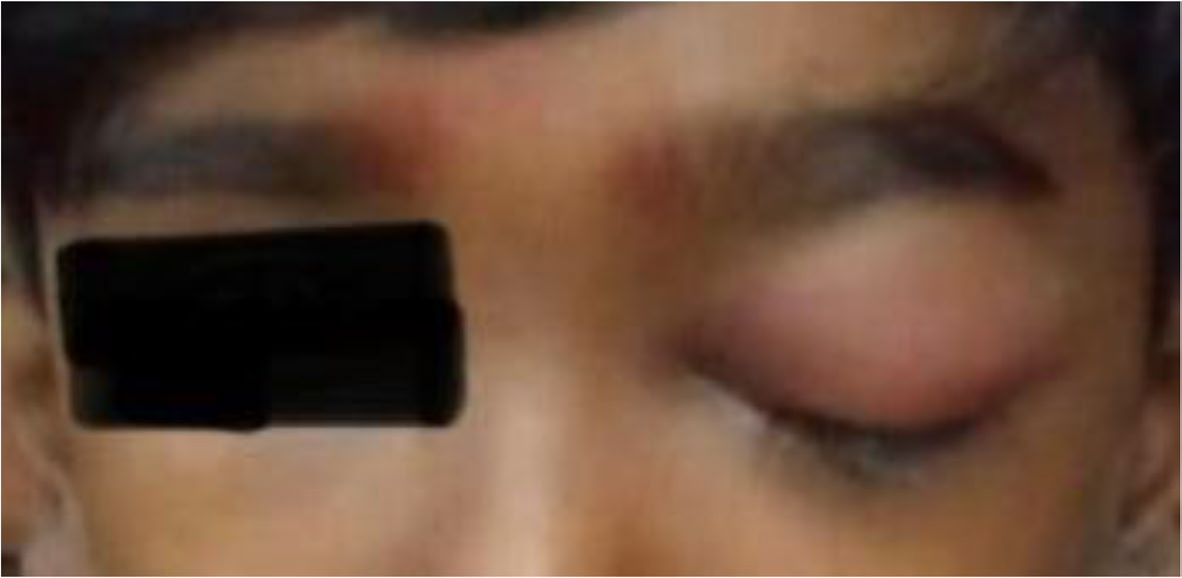
Left peri-orbital swelling

**Fig. 3:**
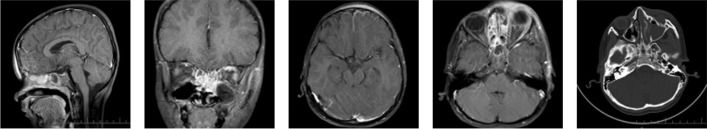
Contrast-enhanced MRI

On the second day of admission, the child started to develop insidious onset, gradually progressing left peri-orbital swelling, tender, appeared red, and proptosis was noted. Vision was intact, but the child could barely open his eyes (Fig. 2). Due to the association of COVID-19 with other co-infections, he was started on amphotericin-B along with meropenem and vancomycin. CT scan revealed complete lumen filling mucosal thickening with slightly hyper dense internal contents noted at left maxillary sinus, bilateral ethmoid sinus, and sphenoid sinus; Mucosal thickening also noted at fronto-ethmoidal recess; mucosal thickening noted along bilateral inferior turbinate, left middle turbinate, and left osteo-meatal complex; mild axial proptosis of the left eye with bulky medial rectus and surrounding fat stranding along with areas of bony erosion noted. Overall features were suggestive of fungal sinusitis with orbital cellulitis. Contrast-enhanced MRI showed lepto-meningeal enhancement noted along left basifrontal lobe convexity, with areas of patchy mucosal necrosis and other features consistent CT findings, overall suggestive of fungal etiology (Fig. 3). Hence, the child was referred to our center for further management.

At the time of admission to our hospital, vitals were stable, weight is 17 kg, length is 122.5 cm, and BMI is 11.4 kg/m^2^ indicating severe thinness as per WHO growth chart. Local examination revealed lemon-sized left peri-orbital swelling. Diagnostic nasal endoscopy was done, and the soft tissue was sent for KOH mount and fungal/bacterial culture. KOH mount did not show any hyphae and culture did not yield any growth. CBC was then done and showed hemoglobin of 11.4, total count 16,500 (neutrophils 70%/lymphocytes 20%), and platelet count of 5.37L. The blood sugar level was 98 mg/dL, glycated hemoglobin was 4.5, and urine ketone bodies were absent. HIV and HbSAg were non-reactive. Inflammatory markers showed C-reactive protein of 72.6 mg/L, serum ferritin of 612.7 nm/mL, lactate dehydrogenase of 358U/L, and D-dimer of 0.04gm/mL. Renal function was within normal limits.

**Fig. 4:**
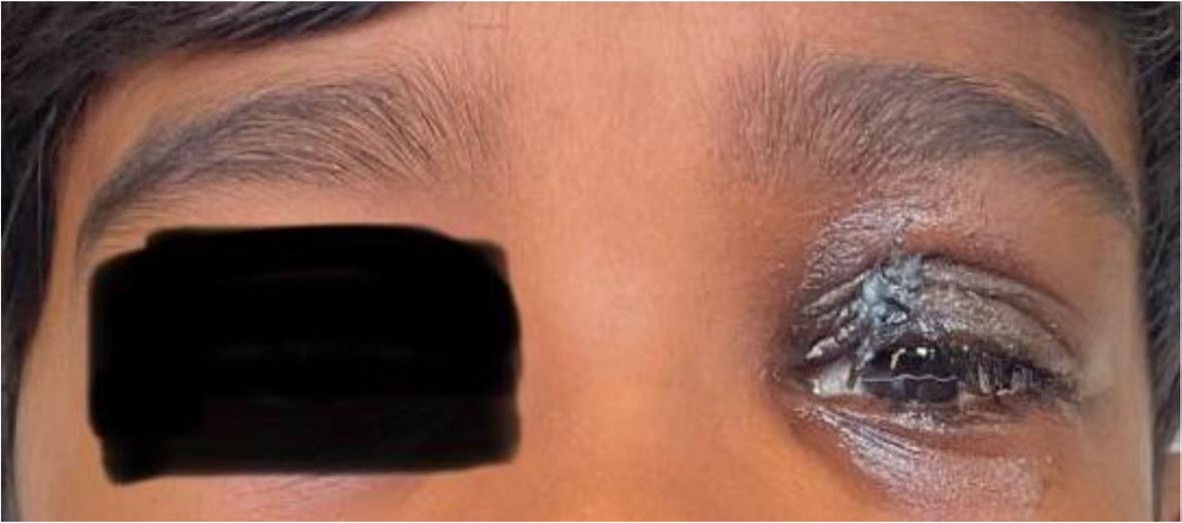
Post-treatment

**Fig. 5:**
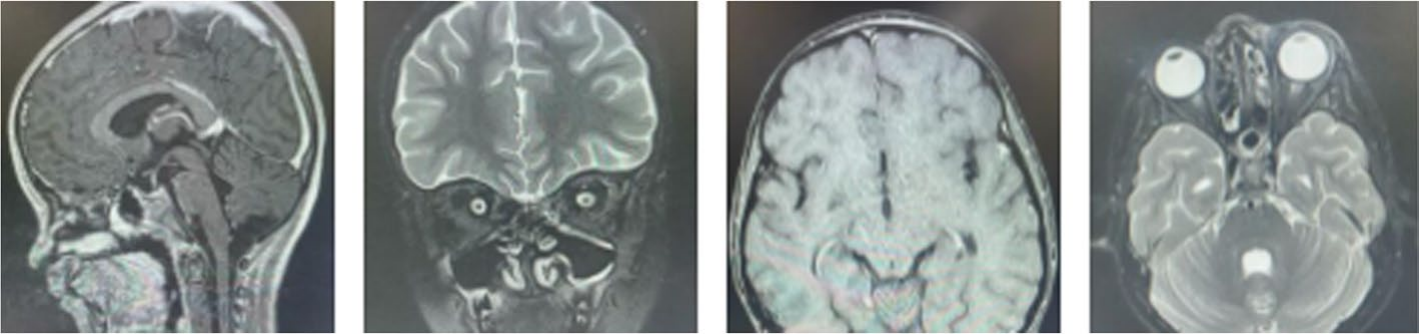
Follow-up MRI

The child was taken up for debridement surgery. Amphotericin B was continued for 22 days. Then, the child developed impaired renal function test. Hence, amphotericin B withheld and started on oral poscoconazole. At the end of 1 month, the child was clinically better with decrease of swelling noticed (Fig. 4). Follow-up MRI was done and showed clear paranasal sinuses, resolution of orbital cellulitis, and regression meningeal enhancement (Fig. 5). He was asked to continue oral poscoconazole for another 14 days and discharged.

Due to known incidence of acute invasive fungal infection associated with coronavirus disease along with radiological imaging suggestive of fungal infection, he was treated as the same and showed significant improvement with early debridement surgery and early institution of anti-fungal therapy.

## Discussion

Severe acute respiratory syndrome coronavirus 2 (SARS-CoV-2) is a novel coronavirus previously unknown to mankind. It is classified as a beta-CoV of group 2B and is the cause of a serious life-threatening disease known as coronavirus disease of 2019 (COVID-19) [10]. SARS-CoV-2 is a virus that is the cause of a serious life-threatening disease known as COVID-19. It was first noted to have occurred in Wuhan, China, in November 2019 and the WHO reported the first case on December 31, 2019. The outbreak was declared a global pandemic on March 11, 2020, and by May 30, 2020 [11].

Beside the acute respiratory distress syndromes (ARDS) caused by it, COVID-19 patients were found to have immune-suppression attributed to a decrease in CD4^+^T and CD8^+^T cells [12]. A complex interplay of factors, including pre-existing diseases, such as diabetes mellitus, previous respiratory pathology, use of immunosuppressive therapy, the risk of hospital-acquired infections, and systemic immune alterations of COVID-19 infection itself may lead to secondary infections, which are increasingly being recognised in view of their impact on morbidity and mortality [13]. Thus, coronavirus disease is known to be associated with several bacterial [10] and fungal co-infections [14]. The main fungal pathogens are Aspergillus, Candida, Mucor, and Cryptococcus [15].

This child had mild COVID-19 illness with severe thinness and no other comorbidities, immunosuppressive state, or any other abovementioned risk factors. The fungus could not be cultured due to early institution of therapy. Similar such clinical and radiological findings have been reported in two adolescents with negative culture reports by Turbin et al. [9].

Acute invasive fungal infection has been recently associated with COVID-19 infection, but this has been in patients with several co-morbidities, receiving high-dose steroids, usually in adult patients. But this is one such rare presentation of a malnourished child with invasive fungal infection in association with COVID-19.

## Conclusion

Acute invasive fungal infection can also occur as an isolated disease in association with COVID-19. We the treating pediatricians should be aware of the possibility of secondary invasive fungal infections in association with COVID-19, and early diagnosis and treatment of this deadly infection might reduce the morbidity and mortality.

## Data Availability

All data and materials available with the authors can be reached on reasonable request.

## Abbreviations

WHO: World Health Organization
RTPCR: Reverse transcription–polymerase chain reaction
ARDS: Acute respiratory distress syndrome
CT: Computer tomography
MRI: Magnetic resonance imaging
BMI: Body mass index
KOH: Potassium hydroxide
CBC: Complete blood count
HIV: Human immunodeficiency virus
HbSAg: Hepatitis b S antigen

## References

[1] Yuen KS (2020). SARS-CoV-2 and COVID-19: the most important research questions. Cell Biosci.

[2] Fang F, Chen Y et al (2020) Chinese Pediatric Society and the Editorial Committee of the Chinese Journal of Pediatrics. Recommendations for the Diagnosis, Prevention, and Control of Coronavirus Disease-19 in Children-The Chinese Perspectives. Front Pediatr 8:553394. 10.3389/fped.2020.553394. 10.3389/fped.2020.553394 PMC7674551 33224906

[3] Schroeder AR, Wilson KM et al (2020) COVID-19 and Kawasaki disease: finding the signal in the noise. Hosp Pediatr; hpeds.2020–000356. 10.1542/hpeds.2020-000356 10.1542/hpeds.2020-000356 32404331

[4] Yang S et al (2021) Bacterial and fungal co-infections among COVID-19 patients in intensive care unit. Microbes Infect;104806. 10.1016/j.micinf.2021.104806 10.1016/j.micinf.2021.104806 PMC7933791 33684520

[5] Nori P (2021). Bacterial and fungal coinfections in COVID-19 patients hospitalized during the New York City pandemic surge. Infect Control Hosp Epidemiol.

[6] Kriengkauykiat J, Ito JI (2011). Epidemiology and treatment approaches in management of invasive fungal infections. Clin Epidemiol.

[7] Tarkan O, Karagün B (2012). Endonasal treatment of acute invasive fungal rhinosinusitis in immunocompromised pediatric hematology oncology patients. Int J Pediatr Otorhinolaryngol.

[8] Singh AK et al (2021) Mucormycosis in COVID-19: a systematic review of cases reported worldwide and in India. Diabetes Metab Syndr. 10.1016/j.dsx.2021.05.019 10.1016/j.dsx.2021.05.019 PMC8137376 34192610

[9] Roger E. Turbin, Peter J, Wawrzusin et al. Orbital cellulitis, sinusitis and intracranial abnormalities in two adolescents with COVID-19. Orbit;39(4):305–310, 10.1080/01676830.2020.1768560 10.1080/01676830.2020.1768560 32419568

[10] Adil MT, Rahman R, et al (2021) SARS-CoV-2 and the pandemic of COVID-19. Postgrad Med J 97(1144):110–116. 10.1136/postgradmedj-2020-138386 10.1136/postgradmedj-2020-138386 PMC10016996 32788312

[11] Adil MT, Rahman R (2021). SARS-CoV-2 and the pandemic of COVID-19. Postgrad Med J.

[12] Anft M, Paniskaki K (2020). COVID-19-induced ARDS is associated with decreased frequency of activated memory/effector T cells expressing CD11a+. Mol Ther.

[13] Chen N, Zhou M (2020). Epidemiological and clinical characteristics of 99 cases of 2019 novel coronavirus pneumonia in Wuhan, China: a descriptive study. Lancet.

[14] Chen N, Zhou M (2020). Epidemiological and clinical characteristics of 99 cases of 2019 novel coronavirus pneumonia in Wuhan, China: a descriptive study. Lancet.

[15] Song G, Liang G (2020). Fungal co-infections associated with global COVID-19 pandemic: a clinical and diagnostic perspective from China. Mycopathologia.

